# Relationship between upward social comparison and financial gambling motives among Chinese lottery gamblers: the roles of relative deprivation and socioeconomic status

**DOI:** 10.3389/fpsyg.2026.1809867

**Published:** 2026-07-20

**Authors:** Huiru Lin, Yue Hu, Jinliang Li, Zhiwei Yang

**Affiliations:** 1 School of Physical Education, Jinan University, Guangzhou, China; 2 School of Management, Jinan University, Guangzhou, China; 3 School of Education and Psychology, Tianjin University of Sport, Tianjin, China; 4 Tianjin Eco-City Affiliated School of Beijing Normal University, Tianjin, China; 5 Guangdong Institute of Smart Education, Jinan University, Guangzhou, China

**Keywords:** financial gambling motives, lottery gamblers, relative deprivation, SES, upward social comparison

## Abstract

This study examined the association between upward social comparison and financial gambling motives among Chinese lottery gamblers, as well as the mediating role of relative deprivation and the moderating role of socioeconomic status (SES) within this relationship. To test this framework, 778 Chinese lottery gamblers (aged 18–79; 568 males and 210 females) completed measures of upward social comparison, relative deprivation, SES, and financial gambling motives anonymously. Results showed that upward social comparison was positively correlated with financial gambling motives, and relative deprivation played a mediating role in this relationship. Additionally, SES moderated both the direct association between upward social comparison and financial gambling motives and the mediating effect of relative deprivation. Specifically, the three sets of associations—between upward social comparison and financial gambling motives, upward social comparison and relative deprivation, and relative deprivation and financial gambling motives—were stronger among Chinese lottery gamblers with a low SES than lottery gamblers with a high SES. These findings contributed to a better understanding of how and when upward social comparison correlates with financial gambling motives.

## Introduction

In recent years, gambling motives have garnered increasing scholarly attention due to their critical association with gambling behaviors and problematic gambling outcomes (Allami et al., 2025; Floyd et al., 2024; Hagfors et al., 2023; Hawker et al., 2026; Lee et al., 2023). A series of empirical studies confirmed that financial gambling motives are closely linked to gambling frequency and at-risk gambling (Hagfors et al., 2022; Magi et al., 2025; Tabri et al., 2022). Qualitative research also pointed out that deprivation-related emotions are strongly correlated with financial gambling motives (Lloyd et al., 2021).

In a meta-analysis of 44 studies, Tabri et al. (2022) noted that money lies at the core of the psychological definition of gambling and that financial gambling motives are moderately associated with both gambling frequency and problem gambling severity. Research based on Chinese samples reveals that financial gambling motives are a key factor linked to gamblers’ transition from rational gambling to problem gambling (Hu, 2019). Identifying the factors associated with financial gambling motives is essential given their adverse correlation with outcomes for gamblers and society. For instance, lottery gamblers with strong financial gambling motives tend to report lower well-being, heavier financial debt, and more deviant behaviors (Li et al., 2022; Komoto, 2025). Given these adverse outcomes, factors correlated with financial gambling motives merit further empirical investigation.

To clarify the research scope, this study strictly distinguishes three core concepts: financial gambling motives, gambling behavior, and problem gambling. Financial gambling motives are defined as internal psychological drives that lead individuals to gamble for economic benefits. As a critical correlational risk factor, they precede excessive gambling and subsequent problem gambling, rather than being identical to gambling behavior or problem gambling.

### Upward social comparisons and financial gambling motives

Upward social comparison describes the tendency for individuals to compare themselves with people who are more advantaged (Festinger, 1954). According to social comparison theory, individuals who focus on others’ advantages and their own perceived shortcomings tend to experience self-doubt, which intensifies negative self-evaluations (Feinstein et al., 2013). Thus, upward social comparison carries more negative connotations than downward social comparison. Studies have also confirmed that upward social comparison is negatively associated with lower self-esteem, resulting in negative emotions such as relative deprivation, anxiety, and depression (Qiu et al., 2017). To protect self-esteem, individuals tend to develop corresponding motives or engage in compensatory behaviors to relieve such negative feelings (Shrum et al., 2013). For example, upward social comparison based on physical appearance is linked to greater investment in personal appearance and physique (Markey and Markey, 2010). Similarly, upward social comparison related to spending power or wealth correlates with higher materialism (Zheng et al., 2018).

By extension, upward social comparison among lottery gamblers is associated with feelings of inferiority and correlates with a weakened positive self-perception. When self-evaluation is threatened, the desire to maintain a positive self-image is linked to compensatory behaviors. In this context, upward social comparison correlates with higher levels of financial gambling motives as a form of compensation. Money and material goods are widely regarded as central parts of personal self-identity. These possessions are correlated with reduced insecurity and stronger feelings of status superiority (Kim et al., 2017; Lee and Shrum, 2011). Additional correlational research documents a consistent link between upward social comparison and financial gambling motives (Zheng et al., 2018). Therefore, we hypothesize that upward social comparison is positively associated with financial gambling motives.

Lottery gambling is the only legal form of gambling in mainland China (Li, 2023) and has a large participant pool. According to La Fleurs’ 2021 World Lottery Almanac, Chinese lottery sales reached approximately $586 billion, ranking first worldwide. A prevalence survey showed that the number of lottery players in China has exceeded 200 million (China Daily, 2012). Furthermore, lottery gambling has unique behavioral features. Different from high-risk gambling activities, lottery participation features low single investment and widespread public participation, making financial gain the dominant motive for most participants. Thus, focusing on lottery gamblers helps reveal the psychological mechanisms underlying financial gambling motives within the Chinese cultural context.

### Upward social comparison, relative deprivation, and financial gambling motives

Relative deprivation refers to the perception that one is disadvantaged compared with others and lacks what one deserves, accompanied by anger and resentment. It is essentially a negative emotional experience (Smith et al., 2012). Over the years, scholars have integrated relative deprivation into social comparison theory (Smith et al., 2012; Xiong and Ye, 2016). According to social comparison theory (Festinger, 1954), gamblers evaluate their own conditions after engaging in upward social comparison. Many gamblers perceive themselves as disadvantaged in this comparison process. This perception generates self-doubt, anger, and resentment. These negative emotions are core features of the subjective experience of relative deprivation. Several empirical studies supported this view and confirmed that upward social comparison positively predicted relative deprivation (Kim et al., 2018; Pan and Zhao, 2024; Xu and Li, 2024).

When gamblers experience relative deprivation, they tend to seek improvements in their financial status. Gambling appears to be a quick and easy way to resolve perceived economic inequities. Accordingly, relative deprivation may motivate gamblers to pursue financial gains through gambling. Indeed, a qualitative study found that viewing gambling as a way to earn money is strongly linked to feelings of deprivation, even though individuals rarely explicitly acknowledge the influence of relative deprivation (Lloyd et al., 2021). Tabri et al. (2015) also found that relative deprivation is positively associated with financial gambling motives among gamblers. Furthermore, emotional motivation theory (Bradley et al., 2001) posits that emotions serve a motivational function, acting as internal drives that activate motivational systems. As a negative emotion stemming from upward social comparison, relative deprivation may correlate with higher financial gambling motives among gamblers. Consistent with this view, prior research has shown that relative deprivation is associated with a stronger desire for immediate monetary rewards (Callan et al., 2011).

Lottery participation has unique advantages in the Chinese context. It involves low monetary costs, no strict participation thresholds, and is widely culturally accepted as a legal form of entertainment. For individuals experiencing relative deprivation, lotteries offer an immediate, low-cost avenue to pursue potential financial gains. In contrast, career advancement requires long-term effort and yields uncertain returns, while excessive consumption can further increase financial strain. Thus, lottery gambling becomes a cognitively accessible and culturally acceptable option for people to ease negative emotions stemming from relative deprivation. In sum, upward social comparison correlates with a stronger sense of relative deprivation, which in turn may be linked to higher financial gambling motives among lottery gamblers.

### Proposed moderated mediation model of upward social comparison and financial gambling motives

To this point, we have proposed that upward social comparison relates to financial gambling motives either directly or indirectly via relative deprivation. Nevertheless, this effect of upward social comparison is not universal; the key lies in whether gamblers possess protective factors against adverse situations.

According to social comparison theory (Festinger, 1954), other factors, such as socioeconomic status (SES), can weaken the associations between upward social comparison and relative deprivation and financial gambling motives. According to the social cognitive theory of social class (Kraus et al., 2012), gamblers with a high SES possess abundant economic and social resources. They have more avenues for self-improvement and tend to adopt positive strategies to alleviate the threat of upward social comparison to self-esteem (Twenge and Campbell, 2002). For this reason, gamblers with a high SES tend to engage in lottery gambling for entertainment or public welfare rather than financial gain, which weakens the correlation of upward social comparison with financial gambling motives.

In contrast, gamblers with a low SES live in more unstable environments, making them more vulnerable to the appeal of large lottery winnings relative to small entry costs (Tabri et al., 2015). For example, they may face dramatic life changes and chronic stress and struggle to access opportunities such as better employment. Compared with high-SES gamblers, they also lack practical ways to improve their financial standing (Hu et al., 2024). Consequently, potential lottery winnings appear to be an effective way for low-SES gamblers to restore positive self-evaluations following upward social comparison. Prior research also indicated that individuals with a low SES may use material values and goals to compensate for discomfort correlated with upward social comparison (Kim et al., 2017). Accordingly, we posit that SES, as a structural social factor reflecting resource disparities, moderates the association between upward social comparison and financial gambling motives.

SES is an objective structural social indicator. Researchers measure SES using three indicators: education, personal annual income, and occupation. Relative deprivation, by contrast, describes a subjective cognitive and emotional experience formed through social comparison. These two constructs operate at distinct analytical levels (Bernstein and Crosby, 1980; Smith et al., 2012). The moderation of SES on the association between upward social comparison and relative deprivation reflects individual differences in subjective emotional arousal when facing the same social comparison stimulus (Mclaughlin et al., 2012). In contrast, the moderation of SES in the paths linking relative deprivation/upward social comparison to financial gambling motives reflects individual differences in behavioral motivation and coping strategies after experiencing negative emotions (Gross, 1998; Neophytou et al., 2023). These are two sequential and independent psychological processes, so including SES as a moderator across multiple paths does not lead to conceptual redundancy. Consistent with the social cognitive theory of social class (Kraus et al., 2012), individuals with different SES differ in both emotional perception and behavioral response patterns.

On the other hand, the degree to which upward social comparison is associated with relative deprivation also differs between high-SES and low-SES gamblers. The social cognitive theory of social class notes that people from different social classes have distinct life experiences and information (Kraus et al., 2012). These differences shape how individuals perceive events and form behavioral tendencies. High-SES gamblers draw on more adaptive cognitive resources and positive emotions during upward social comparison (Bradley and Corwyn, 2002). These resources weaken the association between upward social comparison and feelings of relative deprivation. Mclaughlin et al. (2012) suggested that individuals with a low SES are more likely to report negative self-evaluations and passive cognitive and emotional experiences than those with a high SES. Accordingly, SES moderates the association between upward social comparison and relative deprivation. Specifically, the positive association between upward social comparison and relative deprivation is weaker among high-SES gamblers than among low-SES gamblers.

Furthermore, high-SES gamblers have a rational understanding of the probabilities of winning the lottery. For this reason, when experiencing intense needs such as relative deprivation, they believe they can improve their situation through conventional approaches including investment and professional development, rather than gambling. Consistent with this perspective, prior research notes that individuals with lower socioeconomic standing “may view lotteries as a convenient and otherwise rare opportunity for radically improving their standard of living” (Blalock et al., 2007). Tabri et al. (2015) further found that perceived economic mobility moderates the association between relative deprivation and financial gambling motives. This study therefore hypothesizes that SES moderates the association between relative deprivation and financial gambling motives.

The specific hypotheses of this study are as follows:

*Hypothesis 1*: Upward social comparison is positively associated with financial gambling motives.

*Hypothesis 2*: Relative deprivation mediates the relationship between upward social comparison and financial gambling motives.

*Hypothesis 3*: SES moderates both the direct and indirect paths of the mediation model of “upward social comparison → relative deprivation → financial gambling motives.”

**Figure 1:**
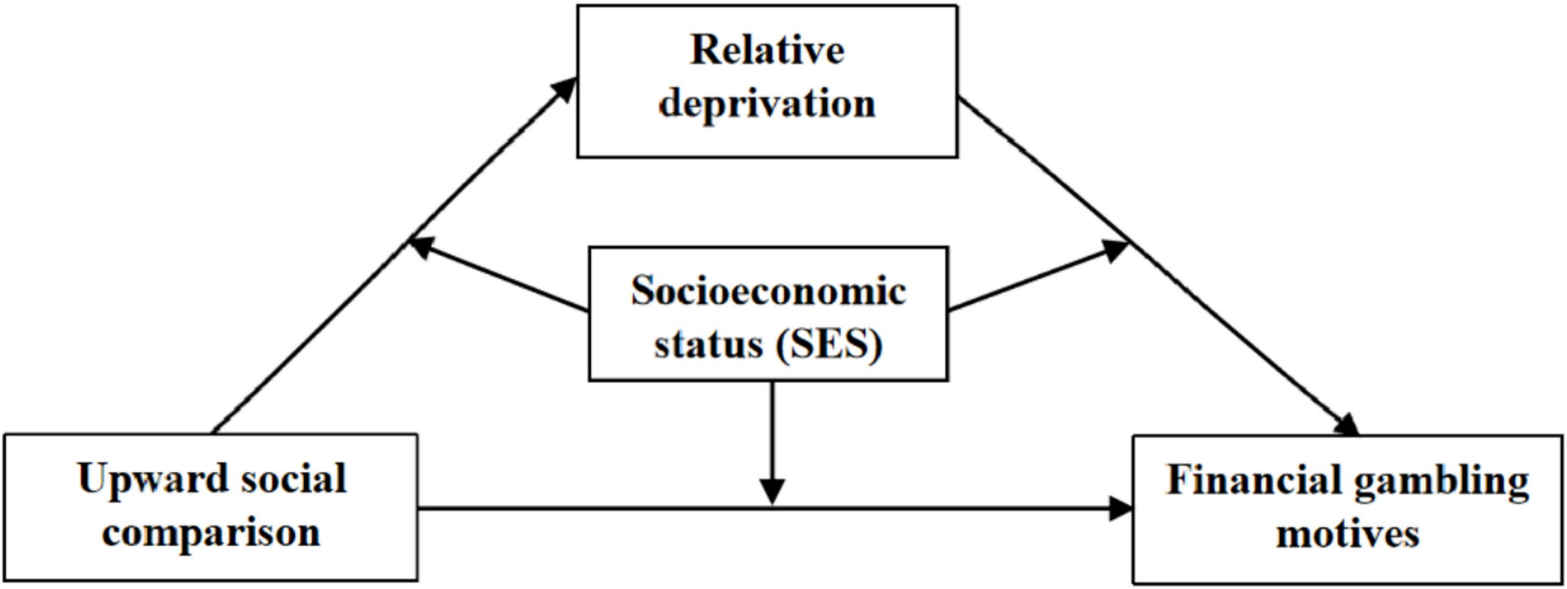
Study model: relative deprivation as mediator and SES as moderator of the relationship between upward social comparison and financial gambling motives.

Overall, the proposed study model is presented in Figure 1.

## Methods

### Participants

Four provinces were selected in this study to guarantee sample representativeness. The sampling sites covered four major regions of China: Fujian (East), Hubei (Central), Sichuan (West), and Jilin (Northeast). In total, 826 Chinese lottery gamblers volunteered to fill out questionnaires. In this study, lottery gamblers were defined as individuals who had purchased lottery tickets continuously for at least 3 months and participated in lottery gambling on a monthly basis (Wang et al., 2021). This operational definition differentiates regular lottery gamblers from occasional players. Since online lottery sales are prohibited in mainland China, we recruited participants from offline lottery retail outlets. Participants completed anonymous on-site questionnaires, and each session lasted 7–10 min. The questionnaires were distributed and collected by the research team. All participants provided informed consent and received three lottery tickets as compensation upon completion of the survey. Once data collection was completed, we screened out invalid questionnaires. Invalid questionnaires refer to those with missed answers, identical responses across all items, or rigid patterned answers (e.g., repeatedly choosing options in the fixed sequence of 1, 2, 3, 4, 5). In total, 778 valid questionnaires were included. The participants ranged in age from 18 to 79 years, with 568 males (73.01%) and 210 females (26.99%). The mean age was 39.85 (SD = 11.97).

### Measures

*Upward social comparison*: upward social comparison was measured using the Chinese version of the Upward Social Comparison Scale (Bai et al., 2013). The scale comprises six items (e.g., “I often compare myself to those who are better than I am”). Gamblers responded on a scale from 1 (*strongly disagree*) to 5 (*strongly agre*e) to the six items. A composite score was calculated by averaging responses across all six items. The internal consistency coefficient for the present sample was 0.84.

*Relative deprivation*: relative deprivation was measured with the Chinese version of the Relative Deprivation Scale (Ma, 2012), which includes four items (e.g., “When I think about what I have compared to others, I feel deprived”). Gamblers were instructed to report whether these descriptions were in line with the four items on a 6-point scale, ranging from 1 (*strongly disagree*) to 6 (*strongly agree*). The total score was obtained by averaging responses across four items. The internal consistency coefficient for the present sample was 0.78.

*Financial gambling motives*: financial gambling motives were measured using the Chinese version of the Gambling Motives Questionnaire-Financial (Hu, 2019), which includes four items (e.g., “I play lottery because I hope to win a big prize by spending a small amount of money”). All items were scored on a 5-point scale ranging from 1 (*almost never*) to 5 (*almost always*). The average of the four items was used as the final composite score. The internal consistency coefficient for the present sample was 0.86.

*SES*: SES was assessed using three indicators: education level, annual income, and occupation. Educational level was coded from 1 (primary school graduate or below) to 6 (postgraduate degree or above). Annual income was coded from 1 (<¥30,000) to 5 (more than ¥200,000). Occupation was scored from 1 to 5 based on China’s vocational qualification standards. Principal component analysis (PCA) objectively derives data-driven weights based on the variance contribution rates of individual indicators. This method not only removes multicollinearity and information redundancy among multidimensional variables but also preserves the maximum amount of valid information from raw data. As a rigorous dimensionality reduction and weighting technique, PCA generates a continuous, accurate, and theoretically robust composite score for SES. Accordingly, we used the framework established by Ouyang and Fan (2018) to calculate the SES composite score in this study. First, the *Z-*scores for the three items were calculated, and then PCA was performed. The score of SES was calculated by the following formula: (*β*_1_*Z_education_ + *β*_2_*Z_occupation_ + *β*_3_*Z_annual income_)/*εf*. Among them, *β*_1_, *β*_2_, and *β*_3_ are the factor loadings of the three items, respectively, and *εf* is the characteristic root of the first factor (Ouyang and Fan, 2018). This PCA-based SES calculation framework has matured and is widely used in previous studies involving Chinese samples (Xu et al., 2024; Ding et al., 2023; Li et al., 2022, 2023). The results of the PCA in this study are as follows: *β1*, *β2*, and *β3* are 0.795, 0.862, and 0.852, respectively; the eigenvalue of the first factor (*εf*) was 1.994; and the first principal component explains 66.481% of the total variance of the three indicators. The higher the score, the higher the SES of gamblers.

### Data analysis

As all data were collected via self-report scales, Harman’s single-factor test was conducted to assess potential common method bias prior to formal statistical analyses. First, descriptive statistics and correlation analyses were computed using SPSS 25.0. Then, SPSS macro-PROCESS (Version 3.5), Models 4 and 59 with 95% bias-corrected confidence intervals (CIs) based on 5,000 bootstrap samples were used to examine the research model. Collinearity diagnostics were performed before statistical analysis. The VIF values of all predictors and interaction terms varied from 1.056 to 1.347, all less than 3, indicating no problematic multicollinearity.

## Results

Harman’s single-factor test was performed to examine common method bias. The variance explained by the first unrotated factor was 29.83%. This value is below the 40% critical threshold (Podsakoff et al., 2003), indicating that common method bias was not a concern in this study.

Descriptive statistics and Pearson’s correlation coefficients for upward social comparison, relative deprivation, financial gambling motives, and SES are presented in Table 1. The correlation matrix showed that upward social comparison was positively associated with financial gambling motives (*r* = 0.34, *p <* 0.001), indicating that upward social comparison was a predictor of financial gambling motives in the sample of Chinese lottery gamblers. Upward social comparison was positively related to relative deprivation (*r* = 0.40, *p <* 0.001). Moreover, relative deprivation was positively related to financial gambling motives (*r* = 0.38, *p <* 0.001).

**Table 1:** Descriptive statistics and correlation matrix of all variables.

Variables	*M ± SD*	1	2	3	4	5	6
1. Gender	1.27 ± 0.44	–					
2. Age	39.85 ± 11.97	–	–				
3. Upward social comparison	3.27 ± 0.83	−0.07	−0.01	–			
4. Relative deprivation	3.72 ± 1.21	0.04	−0.04	0.40^***^	–		
5. Financial gambling motives	2.88 ± 1.15	−0.04	0.01	0.34^***^	0.38^***^	–	
6. SES	0.1 ± 1.00	−0.04	−0.01	−0.07	−0.05	−0.04	–

Gender was dummy-coded (male = 1, female = 2), ^***^p < 0.001 (two-tailed).

Next, Model 4 of the SPSS macro-PROCESS (Version 3.5) was used to test the mediating effect. As a potential mediator, relative deprivation was included in a mediation model to examine whether it mediates the link between upward social comparison and financial gambling motives in Chinese lottery gamblers. Gender and age were taken as control variables, upward social comparison as independent variables, financial gambling motives as dependent variables, and relative deprivation as a mediating variable. All variables in the model were standardized before entering the analysis.

Consistent with Hypothesis 1, the results showed that upward social comparison positively predicted financial gambling motives (*β* = 0.22, *p <* 0.001) in the mediation model. Upward social comparison positively predicted relative deprivation (*β* = 0.41, *p <* 0.001); relative deprivation positively predicted financial gambling motives (*β* = 0.29, *p <* 0.001).

Bootstrap results revealed that the total effect of the mediation model (upward social comparison → relative deprivation → financial gambling motives) was 0.34 (95% CI = [0.27, 0.41]), the direct effect was 0.22 (95% CI = [0.15, 0.29]), and the indirect effect was 0.12 (95% CI = [0.08, 0.15]). The indirect effect accounted for 35.29% of the total effect. These results support Hypothesis 2, confirming that relative deprivation significantly mediates the association between upward social comparison and financial gambling motives.

**Figure 2:**
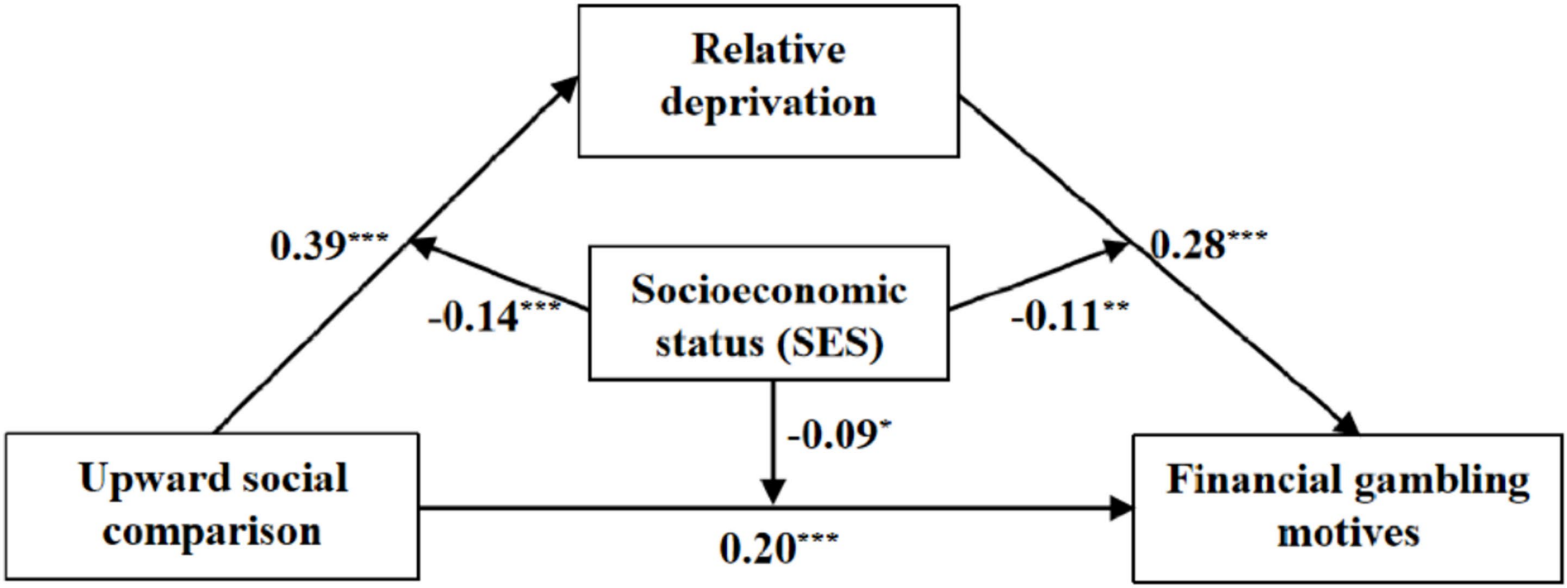
Standardized coefficients of the moderated mediation model. SES moderates three paths in the model.

In addition, Model 59 of the SPSS macro-PROCESS (Version 3.5) was used to test the moderated mediation effect. The results (Table 2) showed that the interaction between upward social comparison and SES significantly predicted relative deprivation (*β* = −0.14, *p <* 0.001) and financial gambling motives (*β* = −0.09, *p* = 0.024); the interaction between relative deprivation and SES significantly predicted financial gambling motives (*β* = −0.11, *p* = 0.003). These findings indicate that SES moderates both the direct and indirect paths of the mediation model (Figure 2). Hypothesis 3 is therefore supported.

**Table 2:** Test of the moderated mediation model.

Variables	*β*	SE	*t*	*p*	95% Confidence interval	
					Lower	Upper
Mediator variable						
Gender	0.06	0.03	1.79	0.073	−0.01	0.13
Age	−0.03	0.03	−0.81	0.419	−0.09	0.13
Upward social comparison	0.39	0.03	11.88^***^	< 0.001	0.33	0.46
Upward social comparison × SES	−0.14	0.04	−3.78^***^	< 0.001	−0.21	−0.07
Dependent variable						
Gender	−0.03	0.03	−0.94	0.349	−0.10	0.03
Age	0.01	0.03	0.40	0.688	−0.05	0.08
Upward social comparison	0.20	0.04	5.60^***^	< 0.001	0.13	0.27
Relative deprivation	0.28	0.04	7.88^***^	< 0.001	0.21	0.35
Upward social comparison × SES	−0.09	0.04	−2.26^*^	0.024	−0.17	−0.01
Relative deprivation × SES	−0.11	0.04	−3.02^**^	0.003	−0.19	−0.04

^*^p < 0.05, ^**^p < 0.01, and ^***^p < 0.001; same interpretation applies below. Standardized regression coefficients were reported.

**Figure 3:**
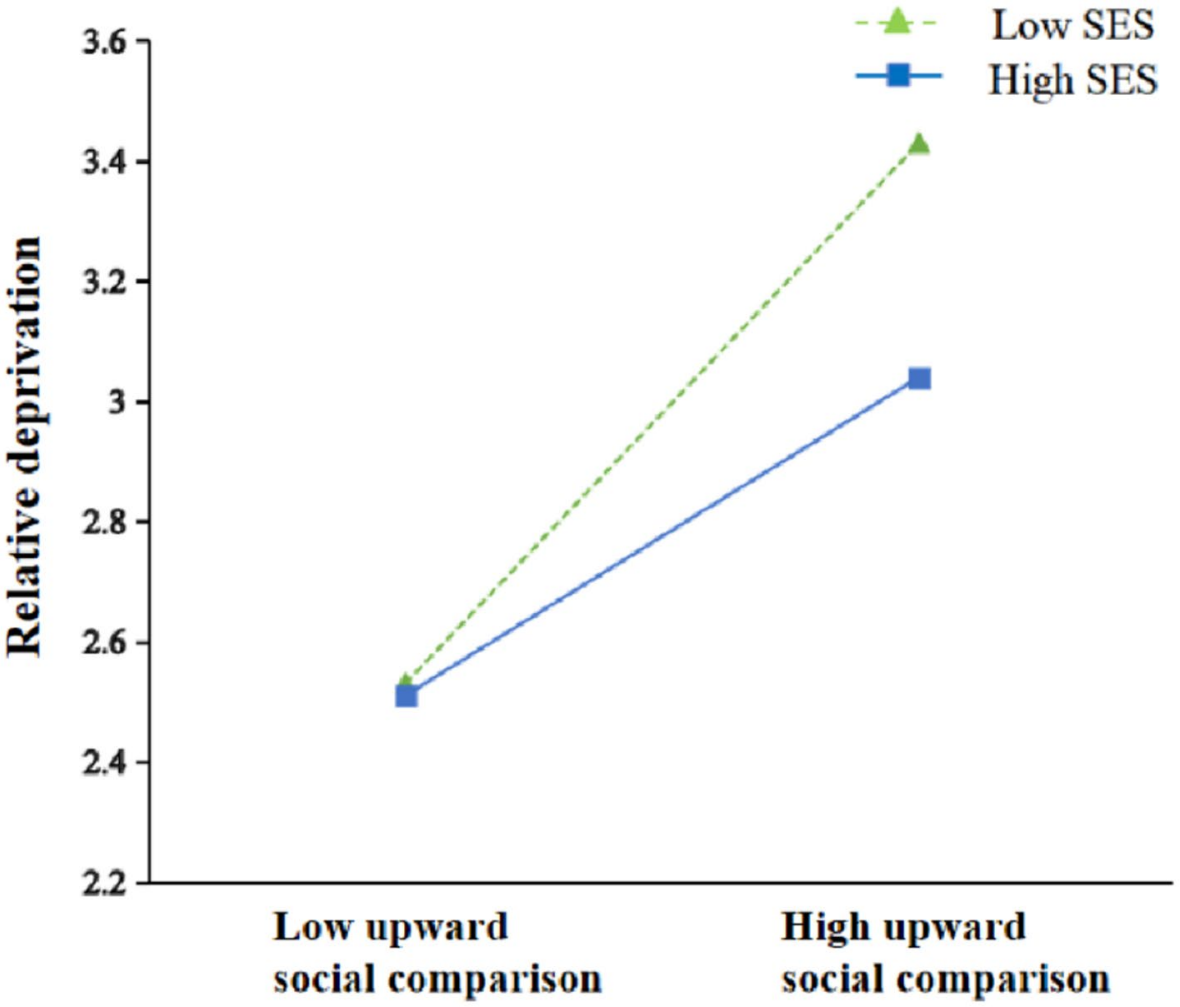
Simple slope plot illustrates the SES’s moderating correlational pattern between upward social comparison and relative deprivation. The green dashed line represents gamblers with a low SES, and the blue solid line represents gamblers with a high SES; the same interpretation applies below. The positive association is stronger for low-SES lottery gamblers.

**Figure 4:**
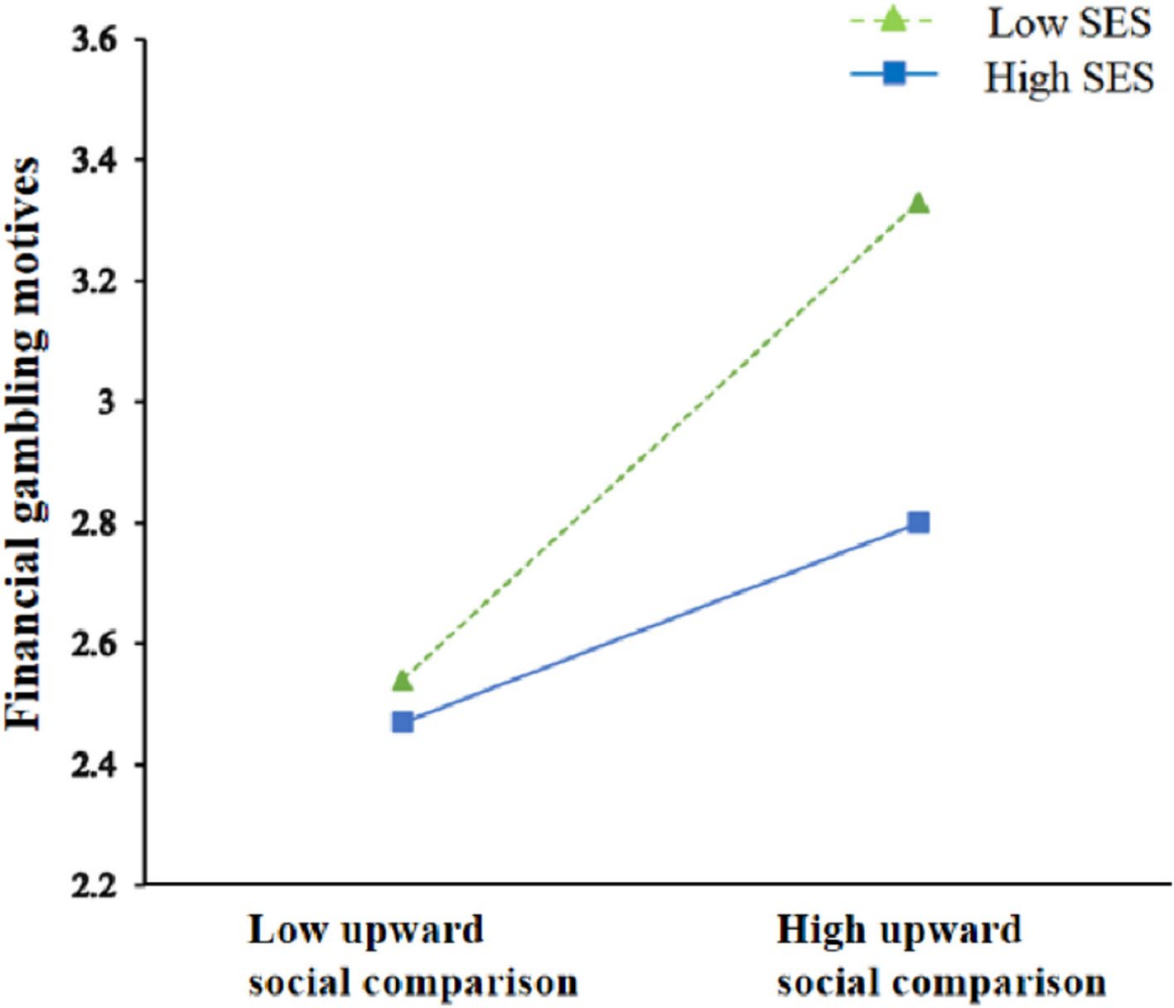
Simple slope plot illustrates SES’s moderating correlational pattern between upward social comparison and financial gambling motives. The positive association is statistically significant among gamblers with low SES, while no significant correlation was detected for gamblers with high SES.

**Figure 5:**
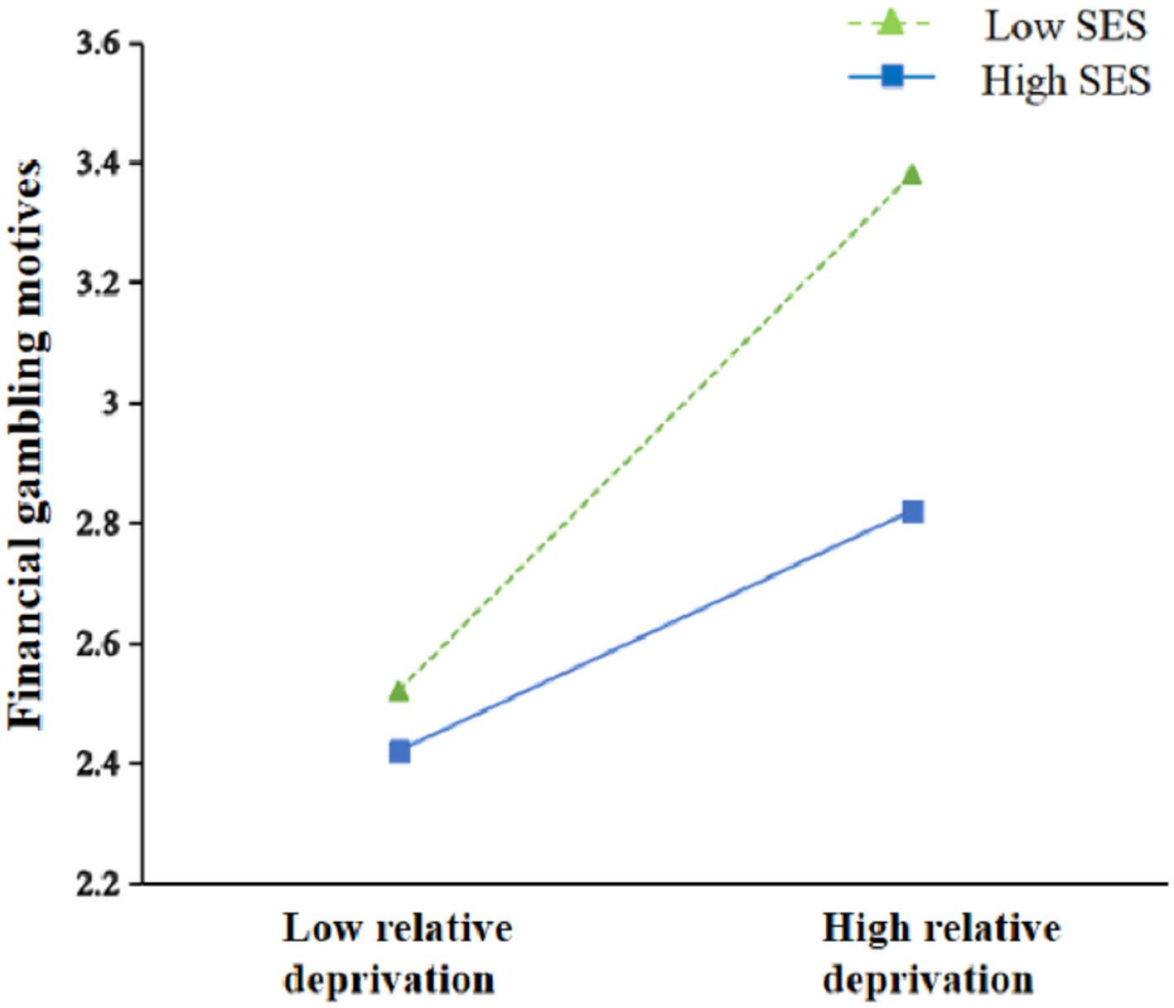
Simple slope plot illustrates SES’s moderating correlational pattern between relative deprivation and financial gambling motives. The positive association is statistically significant among gamblers with low SES, while no significant correlation was detected among gamblers with high SES.

A simple slope analysis was conducted to illustrate the moderating effects of SES. The association between upward social comparison and relative deprivation at 1 standard deviation below and above the mean of SES was examined, respectively. The results (Figure 3) revealed that upward social comparison predicted relative deprivation more strongly among low-SES gamblers (*β* = 0.42, *p <* 0.001) than among high-SES gamblers (*β* = 0.26, *p* = 0.001). The study also separately predicted financial gambling motives against upward social comparison and relative deprivation for gamblers with high and low levels of SES. The results (Figures 4, 5) showed that the associations between upward social comparison, relative deprivation, and financial gambling motives were stronger for low-SES gamblers (*β* = 0.25, *p* = 0.009; *β* = 0.28, *p* = 0.003) than for those with a high SES (*β* = 0.04, *p* = 0.616; *β* = 0.06, *p* = 0.482).

A formal test to compare the conditional indirect effects between low-SES and high-SES gamblers was further conducted. The results showed a significant mediating effect of relative deprivation between upward social comparison and financial gambling motives for low-SES gamblers, a*b = 0.12, 95% CI = [0.02, 0.21], but not for high-SES gamblers, a*b = 0.02, 95% CI = [−0.06, 0.03]. The contrast between the indirect effect of low SES and high SES was [0.10], 95% CI = [0.07, 0.14]. The results confirmed that the indirect effect of relative deprivation differed significantly among gamblers with different SES levels.

## Discussion

Earlier studies have explored the relationships among social comparison, relative deprivation, and gambling motives (Callan et al., 2008, 2011; Tabri et al., 2015). However, few studies have independently treated upward social comparison as a core predictor and systematically examined its correlation with financial gambling motives. Furthermore, this study innovatively incorporated SES as a moderator to construct a moderated mediation model, which supplements existing research in this field.

Consistent with our expectations, upward social comparison among gamblers positively predicts financial gambling motives. This result aligns with social comparison theory (Festinger, 1954) and deepens our understanding of how upward social comparison relates to motives for financial gambling. Most people aim to maintain a sense of personal advantage and avoid inferiority. Unfavorable upward social comparison often disrupts this psychological tendency (Zheng et al., 2018). Lottery gamblers who feel disadvantaged after upward comparison search for ways to lift their socioeconomic position. Many conventional paths to self-improvement require long-term effort and carry uncertain outcomes. In such cases, behaviors driven by financial gambling motives align with their desire to improve personal status (Callan et al., 2008). Given that lotteries offer the potential for instant, substantial financial gains, many gamblers view them as a means to boost their status and wealth and rebuild positive self-perceptions. Existing research supports this viewpoint: materialism—a value system centered on possessions—grows stronger following upward social comparison (Zheng et al., 2018). This study extends the application of social comparison theory (Festinger, 1954) and emotional motivation theory (Bradley et al., 2001) to gambling research.

This study also tests the mediating role of relative deprivation in the link between upward social comparison and financial gambling motives. The results confirm that relative deprivation acts as a mediator between the two variables. Upward social comparison describes the act of evaluating oneself against more privileged others. When people notice others’ material advantages, they often feel inferior and resentful (Zheng et al., 2018). These feelings are accompanied by greater insecurity and relative deprivation. This observed pattern aligns with the core claims of social comparison theory (Festinger, 1954). Prior studies also document that relative deprivation from upward comparison correlates with a greater preference for instant monetary rewards (Callan et al., 2011). As outlined in the Introduction, individuals struggling with relative deprivation are driven to rebuild their sense of deservingness. Many gamblers regard money and possessions as one way to ease insecurity, enhance self-esteem, and relieve feelings of deprivation. For this reason, gambling for profit becomes a quick way to restore a sense of personal deservingness. Higher levels of relative deprivation are accompanied by stronger financial gambling motives. This finding is consistent with existing evidence indicating that relative deprivation positively predicts financial gambling motives (Tabri et al., 2015). It also lends support to emotional motivation theory (Bradley et al., 2001), which posits that relative deprivation is correlated with heightened internal psychological urges toward financial gambling motives. While lotteries rarely deliver instant prizes, the hope of winning and associated psychological rewards can generate positive affects and mitigate negative emotions (Hu et al., 2017). Overall, relative deprivation is an intermediate variable linking upward social comparison to higher financial gambling motives.

However, the associations among upward social comparison, relative deprivation, and financial gambling motives emerge only—or grow much stronger—for gamblers with a low SES. Indeed, moderate analyses of the present data support this view: SES showed statistically significant differences in the strength of the association between upward social comparison and financial gambling motives across both direct and indirect correlational connections. SES shapes how upward social comparison and relative deprivation predict financial gambling motives, instead of directly driving these psychological states. In line with the correlational findings in Table 1, SES shows no significant direct correlations with other variables. Even so, its notable moderating role confirms that SES is a key structural contextual factor in this model.

It is necessary to distinguish the dual roles of SES in this correlational model. We separate these two patterns for clear interpretation. First, as an objective resource variable, SES shapes how individuals perceive social inequality (i.e., relative deprivation) in the face of upward social comparison (Mclaughlin et al., 2012; Smith et al., 2012). Second, SES constrains people’s available coping channels and further regulates the association between negative emotions and financial gambling motives (Gross, 1998; Neophytou et al., 2023). These two stages are distinct and non-overlapping psychological mechanisms, which explains why SES exerts moderating effects on multiple paths simultaneously.

Gamblers with low SES tend to report more structural life constraints and fewer perceived developmental chances. Conventional methods for personal growth rarely align with their goals of raising self-value and economic standing. Relative deprivation correlates closely with perceived unfair economic gaps. Low-SES gamblers show a stronger inclination to seek instant monetary gains to ease deprivation-related negative feelings. Structural barriers to upward mobility remain persistent for this group (Shi and Shen, 2007). Lottery participation shows stronger appeal among low-SES gamblers under these constraints. This correlational pattern aligns with limited developmental opportunities, rather than individual irrationality. In this context, upward social comparison and relative deprivation show far stronger correlations with financial gambling motives. This accounts for the stronger correlational links observed among the three variables in low-SES gamblers. By comparison, high-SES gamblers have abundant resources and are more readily accessible than traditional avenues for improving self-value. Existing studies confirm that higher SES weakens the correlations between risk factors and financial gambling motives (Li et al., 2022). Collectively, these findings indicate that SES, as a fundamental structural social variable, plays a moderating role in the relationships among upward social comparison, relative deprivation, and financial gambling motives.

### Practical implications

In practical terms, the findings provide references to lottery management and responsible gambling advocacy. First, relevant departments should standardize lottery advertising content and reduce exaggerated publicity about huge prizes and wealth-related comparison cues, thereby weakening the trigger for upward social comparison. Second, targeted psychological guidance and popular science activities can be conducted for groups with low SES, helping them learn healthy coping strategies to relieve relative deprivation and establish rational wealth values. Third, community organizations can provide more practical employment and development resources for vulnerable groups, expanding their legitimate channels for income growth and reducing their reliance on lottery gambling for financial expectations.

### Limitations and future research

This study has several notable limitations. First, although we adopted a frequency-based definition to recruit regular lottery participants, the observed variables may partially reflect the psychology of general lottery players rather than pure gambling motives. Future research could adopt more rigorous screening criteria to distinguish different types of lottery players. Second, this study adopted a cross-sectional design, which cannot confirm causal relationships among variables. There may exist reverse correlational patterns: gamblers with stronger financial gambling motives may pay more attention to others’ wealth and thus engage in more frequent upward social comparison. Repeated lottery losses may also coincide with stronger feelings of relative deprivation and more frequent upward social comparisons. In addition, potential third variables (such as individual personality traits and family financial pressure) may interfere with the observed correlations. Future longitudinal and experimental studies are needed to clarify causal relationships among these variables. In addition, subsequent research can explore additional boundary conditions, such as individual values and social atmosphere, and conduct cross-cultural comparisons to examine whether the research model is applicable to gambling groups across different regions.

## Conclusion

This study confirms a positive association between upward social comparison and financial gambling motives and verifies the proposed moderated mediation model. Specifically, relative deprivation mediates the relationship between upward social comparison and financial gambling motives. In addition, SES moderates both the direct association between upward social comparison and financial gambling motives and the mediating effect of relative deprivation. This moderating effect is particularly pronounced among Chinese lottery gamblers with lower SES than in those with higher SES.

## Data Availability

The datasets presented in this article are not readily available because the data that support the findings of this study are available on reasonable request from the corresponding author. The data are not publicly available due to restrictions on their containing information that could compromise the privacy of research participants. Requests to access the datasets should be directed to HL, linhuiru@jnu.edu.cn.
